# Fluorine-Free Hydrophobic Modification and Waterproof Breathable Properties of Electrospun Polyacrylonitrile Nanofibrous Membranes

**DOI:** 10.3390/polym14235295

**Published:** 2022-12-03

**Authors:** Ling Zhang, Junlu Sheng, Yongbo Yao, Zhiyong Yan, Yunyun Zhai, Zhongfeng Tang, Haidong Li

**Affiliations:** 1 Nanotechnology Research Institute, College of Materials and Textile Engineering, Jiaxing University, Jiaxing 314001, China; 2 Key Laboratory of Yarn Materials Forming and Composite Processing Technology of Zhejiang Province, Jiaxing University, Jiaxing 314001, China; 3 Jiaxing Key Laboratory of Molecular Recognition and Sensing, College of Biological, Chemical Sciences and Engineering, Jiaxing University, Jiaxing 314001, China; 4 Shanghai Institute of Applied Physics, Chinese Academy of Sciences, Shanghai 201800, China

**Keywords:** electrospun polyacrylonitrile membranes, fluorine-free hydrophobic modification, amino functional modified polysiloxane, in situ cross-linking reaction, waterproof breathable

## Abstract

Waterproof breathable functional membranes have broad application prospects in the field of outdoors textiles. The fluorine-containing microporous membranes of the mainstream functional products easily cause harm to the environment, and thus, the fluorine-free environmental nanofibrous membranes are an important development direction for functional membranes. In this subject, the electrospun polyacrylonitrile nanofibrous membranes were first hydrophobically modified by amino functional modified polysiloxane (AMP), followed by in situ cross-linking modified with 4, 4’-methyl diphenylene diisocyanate (MDI). The fluorine-free modification by AMP altered the surface of the membranes from hydrophilic to hydrophobic, and greatly improved the waterproof properties with the hydrostatic pressure reaching to 87.6 kPa. In addition, the formation of bonding points and the in situ preparation of polyuria through the reaction between the isocyanate in MDI and the amino group in AMP, could improve the mechanical properties effectively. When using AMP with the concentration of 1 wt% and MDI with the concentration of 2 wt%, the relatively good comprehensive performance was obtained with good water resistance (93.8 kPa), modest vapor permeability (4.7 kg m^−2^ d^−1^) and air permeability (12.7 mm/s). Based on these testing data, the modified nanofibrous membranes had excellent waterproof and breathable properties, which has future potential in outdoor sports apparel.

## 1. Introduction

With the proposal of healthy, green and environmentally friendly lifestyles, outdoor sports have been highly sought after by the general public in recent years, which stimulates the market demand for sports/outdoors textiles to grow sharply [1,2,3]. In order to adapt to the complex and changeable environment of outdoor sports, scientists in academia have done tremendous work in recent years in studying the functional properties, such as waterproofness and breathability, cold resistance, heat preservation or UV protection of the sports/outdoors textiles [4,5,6]. Possessing the functional membranes as the core layer, waterproof and breathable fabric is the representative of the outdoor sports textile products. The hydrophobic microporous membranes contain more holes and interconnected channels, such as polytetrafluoroethylene (PTFE) bidirectional stretch membranes, which make them become the mainstream of the products, but their application is limited by some default properties that make them hard to degrade and easy to enrich [7,8]. Therefore, the development of fluorine-free and environmentally friendly functional waterproof breathable membranes is an important development direction at present.

Electrospinning has prosperous prospect in the field of waterproof breathable membranes because of its unique advantages, such as simple spinning equipment, diversified raw material combinations, strong scalability and controllable spinning process [9,10,11]. Gu et al. [12] fabricated electrospun polyurethane (PU) microporous membranes through changing the volume ratios of the mixture solvents. Furthermore, the resultant membranes presented a modest water vapor transmission rate (WVTR) of 10.1 kg m^−2^ d^−1^, as well as robust mechanical properties with a tensile strength of 6.6 MPa, which is desirable in protective clothing. However, the hydrostatic pressure was only 2.1 kPa and could not meet the actual application requirements [13]. In consideration of improving the waterproofness, the hydrophobic modification of the membranes for waterproof and breathable application has brought about widespread attention [14,15,16]. In order to construct rough structures for acquiring hydrophobicity, Wang et al. [17] prepared PU/SiO_2_ nanofibrous membranes by enriching SiO_2_ particles on the surface. The final polyurethane/SiO_2_ nanofibrous membranes presented a water contact angle (WCA) of 154°, and the hydrostatic pressure reached 8.02 kPa.

To further increase the hydrophobicity as well as the waterproofness of the functional membranes, low surface energy substances including fluorine-containing compounds were selected for hydrophobic finishing [18,19]. Wang et al. [20] fabricated electrospun polyacrylonitrile (PAN) fibrous membrane which was modified with waterborne fluorinated polyurethane (WFPU). After the WFPU modification, the pristine PAN fibrous membranes possessed high waterproofness of up to 83.4 kPa. However, during the use of long-chain fluorocarbon-type finishing agents, some toxic substances are usually left behind. This is because the covalent bond energy of the structure is extremely strong due to the presence of fluorine atoms, and it is not easy for it to decompose in the natural environment [21,22,23]. Therefore, in the selection of finishing agent, the first consideration is to choose short-chain fluorocarbon or fluorine-free finishing agents. Zhang et al. [24] prepared environmentally friendly siliceous polyurethane/stearic acid nanofiber membranes for waterproof breathable application. By adjusting the addition amount of the stearic acid, the waterproofness of SIPU/SA nanofiber membranes was greatly improved with the hydrostatic pressure of 79 kPa. However, the tensile strength was 6.2 MPa and could not meet the needs of practical applications.

**Figure 1:**
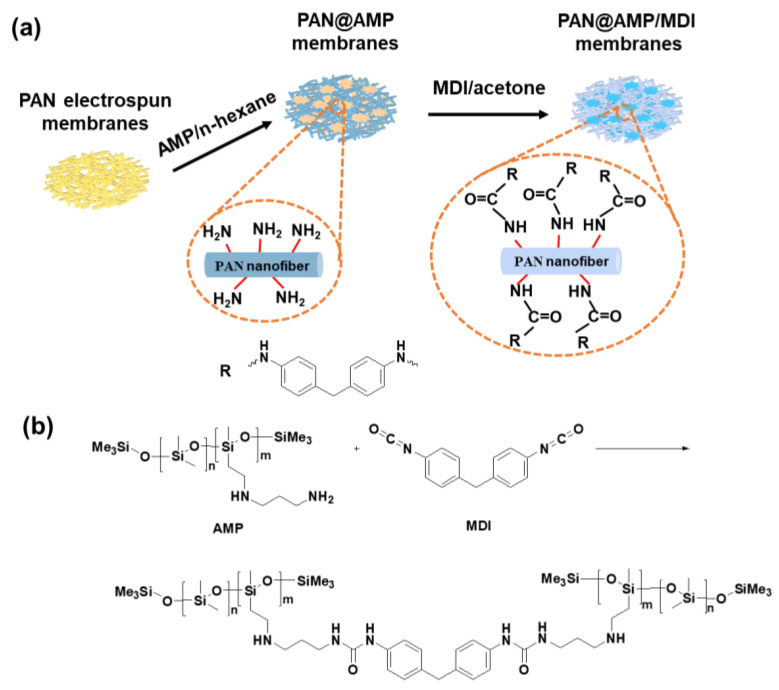
(**a**) Schematic illustration of the preparation procedure of fluoride-free PAN@AMP/MDI nanofibrous membranes. (**b**) The cross-linking reaction between AMP and MDI.

The key to our design was to fabricate composite nanofibrous membranes with robust waterproof and mechanical properties via fluorine-free hydrophobic modification and in situ cross-linking, as illustrated in Figure 1. Due to the small pore diameter, fine tortuous pore channels, high porosity and easy access to be modified, polyacrylonitrile (PAN) nanofibrous membranes were chosen as the backbone for fabricating the functional membrane. After the fluorine-free modification with amino functional modified poysiloxane (AMP), there was hydrophobic adhesion structure interspersed in the nanofibrous membranes, which would not only endow the membranes with hydrophobicity, but also enhance the mechanical performance of the membranes at the same time. In order to further improve the strength of the PAN@AMP membranes, 4, 4’-methyl diphenylene diisocyanate (MDI) were introduced subsequently for the in situ cross-linking reaction with AMP. By adjusting the concentration of AMP and MDI, the effects on the morphology structure, wetting behavior, mechanical property and waterproof breathable performance were investigated. This design may offer a new avenue for the development of a functional waterproof breathable nanofibrous membranes that meet the needs for wide application, such as protective clothing, a separation process, and self-cleaning materials.

## 2. Materials and Methods

### 2.1. Materials

Polyacrylonitrile (PAN, M_w_ = 90,000 g/mol) was acquired from Kaneka Co., Ltd., Tokyo, Japan. Amino functional modified polysiloxane (AMP, surface energy is 20 J m^−2^) and 4, 4’-methyl diphenylene diisocyanate (MDI, M_w_ = 250.25) were supplied from Aladdin Chemical Reagent Co. N, N-dimethylformamide (DMF), acetone, and n-hexane were brought by Sinopharm Chemical Reagent Co. Ltd., Shanghai, China. All reagents were employed as supplied without any processing.

### 2.2. Fabrication of PAN@AMP/MDI Nanofibrous Membranes

The PAN powders were dissolved in DMF and stirred for 12 h to make a uniform and transparent electrospun solution with a concentration of 9 wt%. PAN nanofibrous membranes were then fabricated under a needle electrospinning environment (25 ± 2 °C and 45 ± 2%) through a DXES-3 electrospinning machine (SOF Nanotechnology Co., Ltd., Shanghai, China), while the solution perfusion rate was 1.0 mL/h, the receiving distance was 21 cm and the voltage was 25 kV.

Subsequently, the as-spun PAN nanofibrous membranes were immersed in AMP/n-hexane solutions, where the solution concentrations were 0.5, 1, 2 and 4 wt%, respectively. Thereafter, the modified nanofibrous membranes were dried in an oven at 100 °C for 30 min, so that the hydrophobic agent formed a hydrophobic and stable adhesion structure in the nanofibrous membranes. For the convenience of subsequent representation, the membranes modified with AMP solution with a concentration of x wt% were defined as PAN@AMP-x.

Finally, the PAN@AMP nanofibrous membranes were modified with different concentrations of MDI/acetone solutions through coating treatment, where the solution concentrations were 0.5, 1, 2 and 4 wt%, respectively. Then, the PAN@AMP/MDI nanofibrous membranes were prepared by then being dried in an oven at 100 °C for 30 min for the cross-linking reaction. Relevantly, the PAN@AMP/MDI nanofibrous membranes were defined as PAN@AMP-x/MDI-y, where y is the concentration of MDI/acetone.

### 2.3. Structure Characterization and Performance Measurement

A field emission scanning electron microscope (FE-SEM, Hitachi S-4800, Chiyoda City, Japan) was used to characterize the morphology and structure of the nanofibrous membranes. The pore size and distribution of the nanofibrous membranes were analyzed through the gas permeation pore size analyzer (CFP-1100AX, PMI Inc., Newtown Square, PA, USA). Porosity of the samples was performed using the equation below [25]:

(1) $$Porosity\;=\;\left(1\;-\;\frac{m}{t\;\times \;S\;\times \rho }\right)\;\times \;100\%$$

where *m*, *t*, and *S* are the mass, thickness, and area of per unit measured membrane, respectively. Furthermore, *ρ* is the density of the raw material. A Nicolet 8700 FT-IR spectrometer was used to verify the presence of the modification agents on the modified membranes, and the ATR total reflection mode of the Fourier transform infrared spectrometer was used to characterize the prepared membranes. X-ray photoelectron spectroscopy (XPS, Thermo Fisher Scientific, Escalab 250Xi, Waltham, MA, USA) was utilized to test the surface chemical compositions. The surface roughness of the prepared membranes was obtained by atomic force microscopy (AFM, Dimension Icon, Bruker, Billerica, MA, USA).

An XQ-1C tensile tester (Shanghai New Fiber Instrument Co., Ltd., Shanghai, China) was used to investigate the tensile strength of the as-prepared membranes. The WCA was usually used to characterize the surface wettability of the nanofibrous membranes using an optical contact angle measuring instrument (KRUSS DSA30). Taking the waterproof property into consideration, hydrostatic pressure was measured based on AATCC 127 test criterion with a water pressure increasing rate of 6 kPa min^−1^. The breathable performance of the membranes was determined by the testing water vapor transmission rate (WVTR) and air permeability. WVTR testing was performed according to the ASTM E96 evaporation method standard with the temperature of 38 °C, relative humidity of 50% and a wind velocity of 1 m s^−1^. Following the ASTM D 737 criterion, the air permeable performance was tested under a differential pressure of 100 Pa. For each sample, hydrostatic pressure, WVTR and air permeability were tested at least three times.

## 3. Results and Discussion

### 3.1. Effect of AMP Concentration

#### 3.1.1. Morphology and Structure

**Figure 2:**
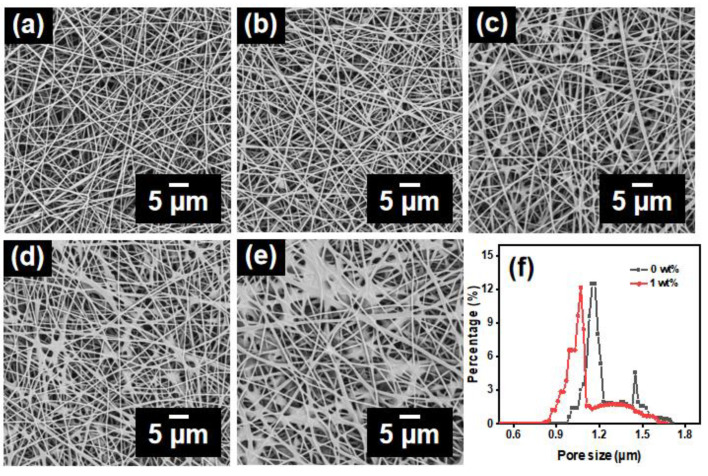
SEM images of hydrophobically-modified PAN@AMP membranes with different concentrations of AMP: (**a**) 0, (**b**) 0.5, (**c**) 1, (**d**) 2 and (**e**) 4 wt%. (**f**) Pore size distribution of PAN original membranes and PAN@AMP-1 membranes.

Due to its small surface tension, AMP can spread easily and form a film on the surface of the PAN nanofibrous membranes, which changes the morphology and surface properties with endowing the hydrophobic properties of the nanofibrous membranes. By comparing the SEM images of PAN@AMP nanofibrous membranes hydrophobically modified by different concentrations of AMP (Figure 2a–e), it can be seen that the untreated PAN nanofibrous membranes have three-dimensional irregular non-woven structures, and the nanofibers are horizontal and vertical with uniform fineness at 318 nm. With the increase of AMP concentration from 0.5 to 4 wt%, there was an increment from 348 to 411 nm of the average fiber diameter (Figure S1a). When the concentration of AMP was 0.5 wt%, some bonding points appear on the nanofibers and are not connected each other. With the increase of AMP concentration, it was obvious that the adhesion structures were gradually formed between nanofibers. When the concentration of AMP was increased continually to 4 wt%, a wide range of film-shaped adhesion structures were observed in the nanofibers, and the adhesion points were connected to each other.

Considering the effect of the film-shaped adhesion structure on the porous structure of the membranes, the pore size distribution of PAN original membranes and PAN@AMP-1 membranes were investigated, as shown in Figure 2f. It was found that the average pore size of the untreated nanofibrous membranes was 1.18 µm. After hydrophobic modification with 1 wt% of AMP, the average pore size of the nanofibrous membranes was significantly reduced, and the pore size distribution was mainly concentrated in the vicinity of 1.07 µm, which was consistent with the phenomenon observed in the relevant SEM images.

#### 3.1.2. Mechanical Properties

**Figure 3:**
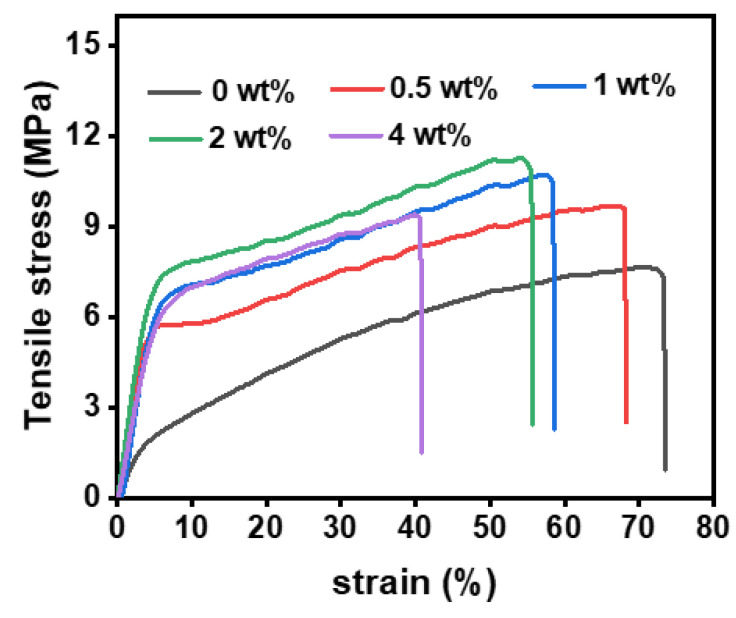
Stress–strain curves of hydrophobically-modified PAN@AMP nanofibrous membranes with different concentrations of AMP.

Considering the practical application of the PAN@AMP membranes, the mechanical performance of the modified membranes was measured. From the stress–strain curves in Figure 3, it can be seen that the strength of the untreated nanofibrous membranes were only 7.7 MPa and the strain was 73.4%. The modification of AMP brought about the obvious promotion of the strength of the PAN@AMP membranes. When the concentration of AMP increased from 0.5 to 2 wt%, the strength increased from 9.7 to 11.3 MPa, which increased by 46% compared with the original membranes. The significant promotion was due to the addition of AMP making the nanofibers stick together, endowing the nanofibers with a certain binding force, and increasing the strength of the nanofibrous membranes to a certain extent. However, the AMP concentration was further increased to 4 wt%, the strength of nanofibrous membranes decreased because of the large area film-shaped adhesion structure coving the PAN membranes, and the strength formed by AMP was poor [26]. Different from the law of strength, the strain always showed a decreasing trend from 73.4% to 40.7%, because the increase of the adhesion point made the nanofibers difficult to move and the strain decreased.

#### 3.1.3. Waterproof Breathable Performance

**Figure 4:**
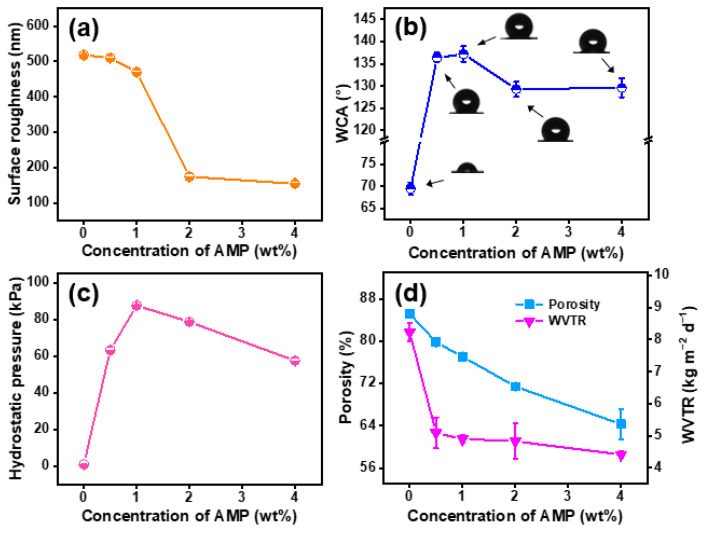
Surface wettability and waterproof breathable permeability of hydrophobically-modified PAN@AMP membranes with different concentrations of AMP: (**a**) Ra values, (**b**) contact angle, (**c**) hydrostatic pressure, (**d**) porosity and WVTR.

In addition, in order to study the effect of AMP concentration on the wettability of the modified membranes surface, the static and dynamic wetting behaviors were tested. As presented in Figure 4a, the calculated Ra values of the PAN, PAN@AMP-1, PAN@AMP-2 and PAN@AMP-4 membranes were 518, 510, 470, 174 and 154 nm, respectively. The decreasing Ra values confirmed the decreased surface roughness of modified PAN@AMP membranes towards the AMP increasing. When tested with static water droplets, as a result of the existence of polarity cyanogroup, the untreated PAN nanofibrous membranes had the certain hydrophilicity with 69.5° of the contact angle. After the modification of AMP as low surface energy substances, the surface property was changed from hydrophilic to hydrophobic, and the WCA of PAN@AMP-0.5 membranes was 136.4°. When further increasing the concentration to 1 wt%, the WCA increased to 137.2°. However, when the concentration of AMP continued to increase to 2 wt%, the film-shaped adhesion structure resulted in the reduction of the surface roughness of the membranes, the WCA decreased to 129.3°, and remained unchanged at the concentration of 4 wt% (129.6°). This phenomenon was the synergistic effect between the low surface energy and rough surface.

When the dynamic water method was used for the test, the nanofibrous membranes gradually became wet under a certain period of time, and were generally characterized by the hydrostatic pressure, as shown in Figure 4c. The PAN original nanofibrous membranes were wetting quickly due to their good hydrophilicity, and the hydrostatic pressure was only 1.1 kPa. While with the introduction of AMP, the hydrostatic pressure of the PAN@AMP nanofibrous membranes increased obviously. When the concentration was 1 wt%, the hydrostatic pressure increased to 87.6 kPa, which was caused by the increase of the contact angle and the decrease of the pore size of the nanofibrous membranes. When the concentration was further increased, the hydrostatic pressure decreased, and it dropped to 57.5 kPa when the concentration was 4 wt%. This was because the morphology and structure of the nanofibrous membranes were affected when the AMP content was too high, the surface roughness was reduced resulting in the decrease of the contact angle, and the hydrostatic pressure of the hydrophobically-modified nanofibrous membranes was reduced accordingly.

Meanwhile, the influence of different concentrations of AMP on the porosity and WVTR of the nanofibrous membranes was also explored, as shown in Figure 4d. The porosity of the original PAN membranes was 85.2% and decreased greatly with the increase of AMP concentration. When the concentration increased to 4 wt%, the porosity decreased to 64.3%. This decreasing trend was due to the introduction of AMP, which gradually produced a wide range of film-shaped adhesion structures on the surface of the nanofibrous membranes leading to the reduction of pores between nanofibers. Since the untreated nanofibrous membranes have good hydrophilicity and are easily wetted by water, the positive cup method was used to measure the WVTR of the original PAN and the PAN@AMP nanofibrous membranes. As shown in Figure 4d, it was found that the WVTR of the original membranes was 8.2 kg·m^−2^·d^−1^, and with the increase of AMP content, the WVTR showed a continuous downward trend. Finally, as the concentration increased to 4 wt%, the WVTR decreased to 4.4 kg·m^−2^·d^−1^, which was consistent with the change trend of porosity.

Comparing these testing data, the treatment with 1 wt% AMP could give the best comprehensive performance of the nanofibrous membranes with the strength of 10.7 MPa, the strain of 58.2%, the contact angle of 137.2°, the hydrostatic pressure of 87.6 kPa and a WVTR of 4.9 kg·m^−2^·d^−1^. Therefore, PAN@AMP-1 was selected as the substrate in subsequent experiments to study the effects of different concentrations of MDI on the structure and performance of nanofibrous membranes.

### 3.2. Effect of MDI Concentration

#### 3.2.1. Morphology and Structure

**Figure 5:**
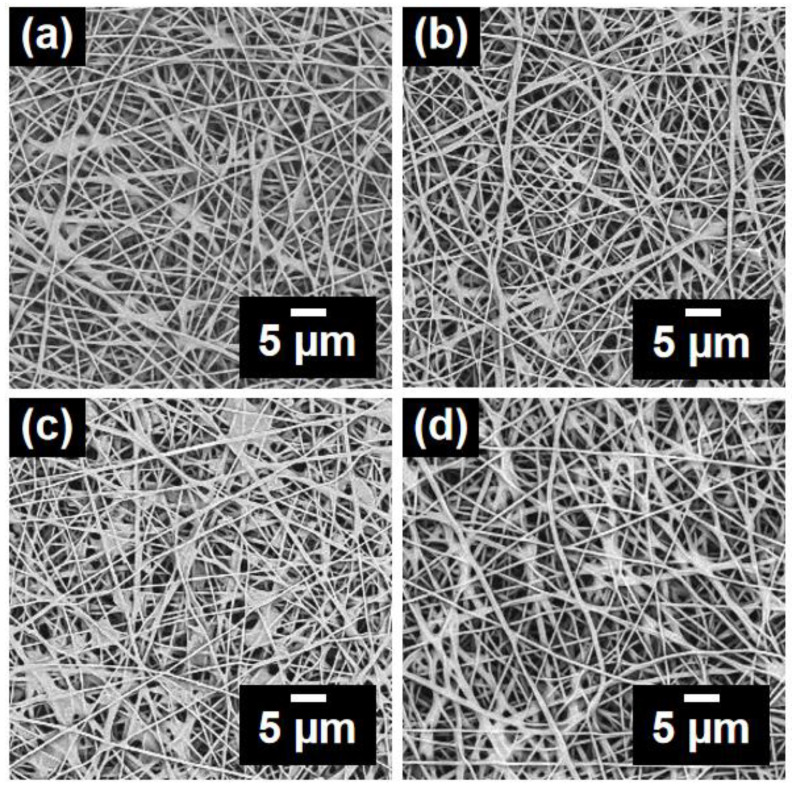
SEM images of PAN@AMP-1/MDI nanofibrous membranes modified by in situ cross-linking of MDI with different concentrations: (**a**) 0.5, (**b**) 1, (**c**) 2 and (**d**) 4 wt%.

It was proof-of-concept and reported in the previous literature that isocyanate (–NCO) in MDI may react with amino (–NH) in AMP to generate new substances under certain conditions [27]. To confirm this, PAN@AMP-1 was selected as the substrate for being modified through introducing MDI. As seen in Figure 5, after the introduction of MDI into the PAN@AMP-1 nanofibrous membranes, the adhesion structure slightly increased with the increase of MDI concentration. Moreover, it can be seen that most of the newly added MDI were captured on the film-shaped adhesion structure formed by AMP, facilitating the in situ cross-linking reaction between MDI and AMP.

#### 3.2.2. FT-IR Spectral Characterization, XPS Analysis and Mechanical Properties

**Figure 6:**
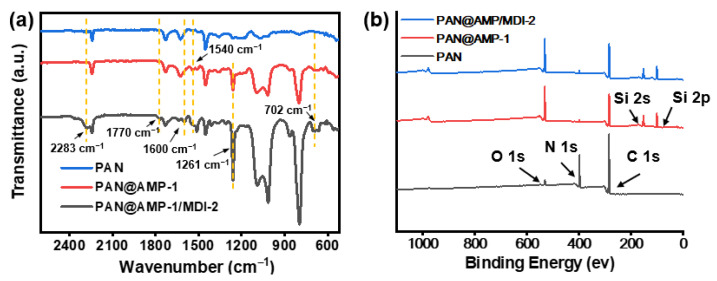
(**a**) FT-IR spectra and (**b**) XPS survey spectra of PAN original membranes, PAN@AMP-1 and PAN@AMP-1/MDI-2 nanofibrous membranes.

As shown in Figure 6a and Figure S2, only a typical stretching vibration absorption peak of cyanogroup (C≡N) was observed at 2243 cm^−1^ in regard to the PAN original nanofibrous membranes. The new peaks of 1259 cm^−1^, 1089 cm^−1^ and 1014 cm^−1^ appeared in the PAN@AMP-0.5 nanofibrous membranes in the Figure S2. The bending vibration absorption peak of C-H in Si-CH_3_ is 1259 cm^−1^, and the double peaks at 1089 cm^−1^ and 1014 cm^−1^ are the characteristic peaks of the asymmetric stretching vibration of Si-O-Si group [28,29], which indicated that AMP was indeed attached to the nanofibrous membranes. Furthermore, the intensity of the three peaks was also enhanced with the increase of AMP concentration. Besides, the characteristic peak of the –NH group in AMP was at 1540 cm^−1^, which was not very obvious due to the relatively small content of amino in AMP.

In order to verify the in situ cross-linking reaction between MDI and AMP, the infrared spectra of PAN original membranes, PAN@AMP-1 nanofibrous membranes and PAN@AMP-1/MDI-2 membranes were analyzed in Figure 6a. The analysis showed that the characteristic peak of –NCO group in MDI was observed at 2283 cm^−1^ in the PAN@AMP-1/MDI-2 nanofibrous membranes, indicating that MDI was indeed attached to the PAN@AMP-1/MDI-2 nanofibrous membranes. The characteristic peak of –NCO group at 2283 cm^−1^ suggested that MDI was not fully involved in the cross-linking reaction. Besides, the characteristic peak of the -NH group in AMP only existed in PAN@AMP-1 nanofibrous membranes at 1540 cm^−1^. After the introduction of 2 wt% MDI, the characteristic peak of the –NH group in AMP disappeared, and a new characteristic peak appeared at 1600 cm^−1^, which was the stretching vibration absorption peak of the carbonyl group (–C=O) of the urea structure. The absorption peak of the amine group of the urea structure was 1317~1261 cm^−1^, and 702 cm^−1^ was the bending vibration absorption peak of –NH. The alteration of the spectra indicated that the –NCO group in MDI and the –NH group in AMP indeed underwent in situ cross-linking reaction and generated polyurea. In addition, due to the strong activity of MDI, some monomers tended to self-polymerize into dimers [30]. The characteristic peak at 1770 cm^−1^ in Figure 6a was the stretching vibration absorption peak of the carbonyl group (–CO) of MDI dimers.

Figure 6b exhibited the typical XPS survey spectra of the PAN original membranes, PAN@AMP-1 and PAN@AMP-1/MDI-2 nanofibrous membranes. For the PAN original membranes, only carbon, nitrogen and oxygen were observed. The intensity of nitrogen in PAN@AMP-1 membranes was reduced due to the adhesion structures interspersing into the nanofibrous membranes. In order to verify the stability of the composition of the PAN@AMP-1 nanofibrous membranes depends on the interaction of PAN and AMP, XPS analysis of the samples before and after breathability tests using different pressures were investigated. Table S1 exhibited the atomic ratios of the samples before and after breathability tests using different pressures at 100 Pa and 200 Pa, respectively. From the results, there was no significant change of the atomic ratios of carbon, nitrogen, oxygen and silicon of the relevant membranes, which verified the stability of the composition of the composite nanofibrous materials.

Subsequently the modification of MDI increased the intensity of nitrogen in PAN@AMP-1/MDI-2 nanofibrous membranes, which were constant with the measured compositions for those samples, as summarized in Table 1. In order to further define the surface coverage, the details of the high-resolution N 1s peak were presented in Figure S3. The PAN original membranes exhibited a peak at 399.2 eV, which are assigned to –C≡N group [31,32]. After being modified by AMP with the adhesive structure on the surface of the PAN membranes, the peak area of –C≡N group at 399.2 eV was obviously smaller than that of the PAN original membranes. As shown in Figure S3b, PAN@AMP-1 nanofibrous membranes demonstrated the binding energies at about 399.9 eV, which is attributed to –C–N group; the result was in good agreement with that previously reported [33]. PAN@AMP-1/MDI-2 membranes presented the peaks at 399.9 and 400.9 eV, which were assigned to –C–N group and amide group (–CONH–), which was consistent with the FT-IR results (Figure 6a).

**Figure 7:**
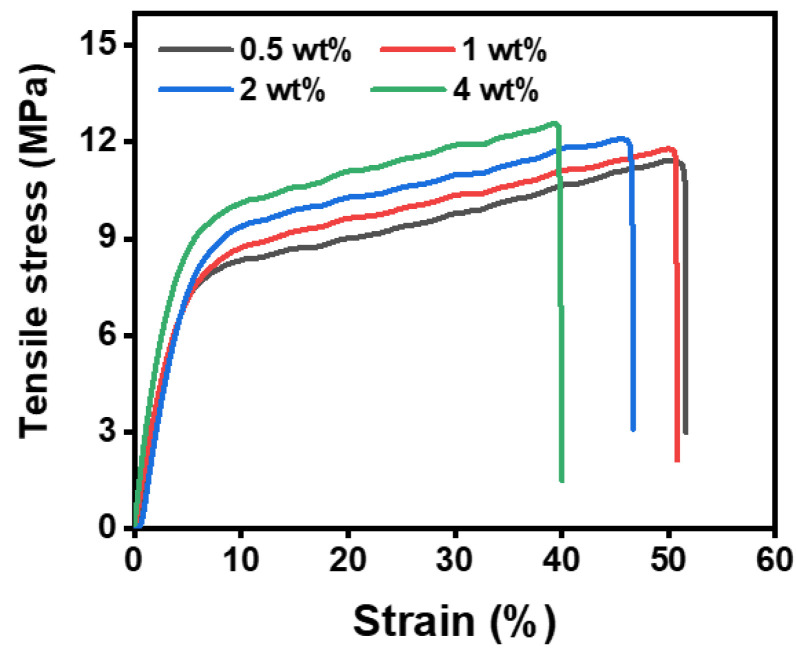
Stress–strain curves of PAN@AMP-1/MDI nanofibrous membranes modified with different concentrations of MDI.

Based on FT-IR and XPS analysis, polyurea was generated by an in situ cross-linking reaction between isocyanate components and amino compounds, which has good strength and wear resistance [34]. Therefore, the influence of different MDI contents on the mechanical properties of PAN@AMP/MDI membranes were investigated, as shown in Figure 7. From Figure 3, the tensile stress of PAN@AMP-1 nanofibrous membranes was 10.7 MPa, and the strain was 58.2%. Comparing with the mechanical property of PAN@AMP-1 nanofibrous membranes, the modification of MDI improved the tensile strength, while on the contrary the strain decreased. When the MDI concentration increased to 4 wt%, the strength increased to 12.7 MPa, and the strain decreased to 39.2%. Compared with the PAN@AMP-1 nanofibrous membranes, the strength increased by 19%, indicating that the introduction of MDI had a certain improvement in the tensile stress of nanofibrous membranes, which is mainly due to the production of polyurea which increased the strength of the modified nanofibrous membranes. The decreasing trend of the strain was because the adhesion structure limited the sliding of the nanofibers in the membranes.

#### 3.2.3. Pore Structure, Surface Wettability and Waterproof Properties

**Figure 8:**
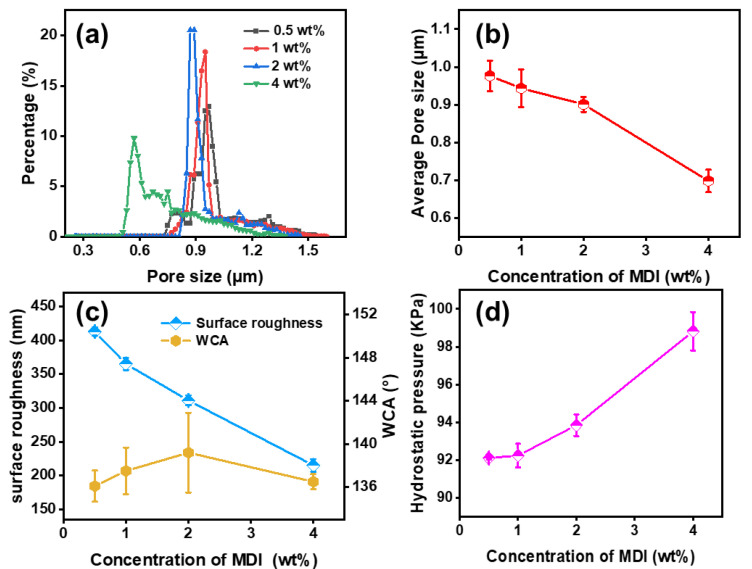
Pore structure, surface wettability and waterproof properties of PAN@AMP-1/MDI membranes modified by in-situ cross-linking of MDI with different concentrations: (**a**) pore size distribution, (**b**) average pore size, (**c**) Ra values and contact angle, (**d**) hydrostatic pressure.

After the apparent morphology of the PAN@AMP/MDI membranes was observed by SEM and their chemical structure was observed by infrared spectra, the microscopic pore structure of nanofibrous membranes was subsequently studied, as shown in Figure 8a and b. In the test of the previous system, it was found that the pore size distribution of PAN@AMP-1 was in the range of 0.81–1.66 μm, with an average pore size of 1.07μm. But after in situ cross-linking with MDI, the pore size of the nanofibrous membranes decreased obviously. Increasing the MDI concentration from 0.5 wt% to 4 wt%, the average pore size decreased from 0.98 μm to 0.70 μm, and the region of concentrated pore size distribution moved towards smaller pore size. 

If the as-prepared membranes could be truly applied to the outdoor field, waterproofness is an essential property. In order to study the waterproof performance of the PAN@AMP-1/MDI nanofibrous membranes, the static contact angle and Ra values were measured, as shown in Figure 8c. In the case of increasing MDI content, the Ra values of the nanofibrous membranes surface were reduced from 413 to 215 due to the slightly increased adhesion structure, which was constant with the morphology observation (Figure 5). However, the value of the contact angle remained basically unchanged in a range of 136.1°–139.2°, which indicated that the low surface energy provided by the AMP with the concentration of 1 wt% conducted the main function. Thereafter, the hydrostatic pressure of the modified membranes was measured by the dynamic hydrostatic pressure method, as shown in Figure 8d. When the concentration of MDI increased, the hydrostatic pressure of the nanofibrous membranes also showed an increasing trend, increasing from 87.6 kPa of PAN@AMP-1 nanofibrous membranes to 98.8 kPa of PAN@AMP-1/MDI-4 nanofibrous membranes. This alteration was resulted from the combined effect of the pore size and surface wettability of the nanofibrous membranes. After the in situ cross-linking modification by MDI, the pore size of the nanofibrous membranes was reduced, so that the hydrostatic pressure was increased to a certain extent.

#### 3.2.4. Moisture and Air Permeability

**Figure 9:**
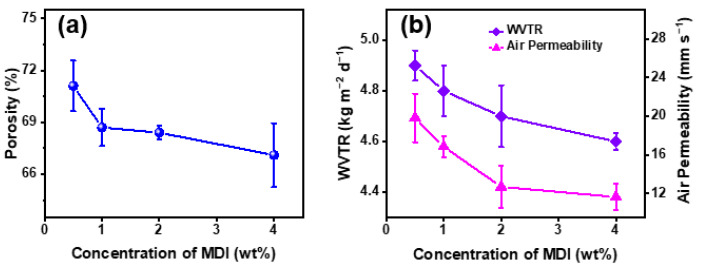
Moisture and air permeability of PAN@AMP-1/MDI nanofibrous membranes modified by in situ cross-linking of MDI with different concentrations: (**a**) porosity, (**b**) WVTR and air permeability.

In addition, moisture and air permeability are also the necessary characteristics of outdoor sports clothing. Since the change of porosity can cause the change of the moisture and air permeability of the nanofibrous membranes, it is necessary to test the porosity of the modified membranes, as shown in Figure 9a. With the increase of MDI concentration from 0.5 wt% to 4 wt%, the porosity decreased slightly from 71.1% to 67.1%.

Generally speaking, WVTR and air permeability are usually used to characterize the moisture and air permeability of nanofibrous membranes. As shown in Figure 9b, with the increase of MDI content, the air permeability presented a decreasing trend from 19.8 mm/s to 11.6 mm/s, which was consistent with the change of porosity. The decreasing trend of porosity also resulted in the WVTR reduction. The WVTR of PAN@AMP-1 nanofibrous membranes was 4.9 kg·m^−2^·d^−1^ and decreased to 4.6 kg·m^−2^·d^−1^ after being modified with the concentration of MDI at 4 wt%.

The above experiments showed that the introduction of MDI content improved the hydrostatic pressure and stress to a certain extent, but the air permeability and WVTR decreased. Comprehensively, it was considered that the MDI concentration of 2 wt% had the best performance, with the tensile stress of 12.1 MPa, the contact angle of 139.2°, the hydrostatic pressure of 93.8 kPa, the WVTR of 4.7 kg·m^−2^·d^−1^ and the air permeability of 12.7 mm/s. Referring to the GB/T 40910-2021 standard “Evaluation of waterproof and breathable properties of textiles”, PAN@AMP-1/MDI-2 nanofibrous membranes have excellent waterproof and breathable properties and were expected to be applied in the outdoor clothes field.

## 4. Conclusions

In this study, the electrospun PAN nanofibrous membranes were modified by AMP and MDI with fluorine-free hydrophobic modification and in situ cross-linking modification. Through performing the morphology structure and pore structure observation, mechanical properties, surface wettability and waterproof breathable performance of nanofibrous membranes, the effects of different concentrations of AMP and MDI were explored, so as to acquire waterproof breathable functional membranes with excellent performance. Firstly, after the hydrophobic modification of different concentrations of fluorine-free hydrophobic agents, a wide range of film-shaped adhesion structures were established among the nanofibers, and the adhesion points were connected to each other, so as to improve the waterproofness and strength of the membranes effectively. Considering the comprehensive performance, PAN@AMP-1 modified membranes were selected for the subsequent in situ cross-linking reaction via MDI modification. Subsequently, through the FT-IR spectrum analysis, it was found that the –NCO group in MDI reacted with the –NH group in AMP to form polyurea. When the MDI concentration was 2 wt%, the prepared PAN@AMP-1/MDI-2 membranes presented the relatively good property with the hydrostatic pressure of 93.8 kPa, the moisture permeability of 4.7 kg·m^−2^·d^−1^, the air permeability of 12.7 mm/s and the strength of 12.1 MPa, which indicated that the as-prepared membranes have extensive application prospects in the fields of waterproof breathable textiles.

## Figures and Tables

**Table 1:** Atomic ratios of carbon, nitrogen, oxygen and silicon on the surface of PAN, PAN@AMP-1 and PAN@AMP-1/MDI-2 nanofibrous membranes. Data are calculated from XPS.

Samples	Atomic Percent (%)			
	C	O	N	Si
PAN nanofibrous membranes	75.31	3.55	21.13	-
PAN@AMP-1 nanofibrous membranes	52.38	23.48	2.16	21.98
PAN@AMP-1/MDI-2 nanofibrous membranes	54.27	22.34	3.51	19.89

## Data Availability

Not applicable.

## Supplementary Materials

The following supporting information can be downloaded at: https://www.mdpi.com/article/10.3390/polym14235295/s1, Table S1: Atomic ratios of carbon, nitrogen, oxygen and silicon on the surface of PAN@AMP-1 nanofibrous membranes before breathability tests, PAN@AMP-1 nanofibrous membranes after breathability tests using pressures of 100 Pa, and PAN@AMP-1 nanofibrous membranes after breathability tests using pressures of 200 Pa. Data are calculated from XPS; Figure S1: (a) Fiber diameters of PAN@AMP nanofibrous membranes modified with different concentrations of AMP. (b) Fiber diameters of PAN@AMP/MDI nanofibrous membranes modified with different concentrations of MDI; Figure S2: FT-IR spectra of hydrophobically modified PAN@AMP nanofibrous membranes with different concentrations of AMP; Figure S3: High-resolution XPS N1s spectra of (a) PAN original membranes, (b) PAN@AMP-1 nanofibrous membranes, and (c) PAN@AMP-1/MDI-2 nanofibrous membranes.
