# Supplementary material for: Fluorine-Free Hydrophobic Modification and Waterproof Breathable Properties of Electrospun Polyacrylonitrile Nanofibrous Membranes
Source: Polymers (Basel). 2022 Dec 3;14(23):5295. doi: 10.3390/polym14235295 (PMC9735966; doi:10.3390/polym14235295)
Supplement: Supplementary file 1 [file polymers-14-05295-s001.zip › polymers-1973513-supplementary.pdf]

Supporting Information for

Fluorine-free Hydrophobic Modification and Waterproof Breathable Properties of Electrospun Polyacrylonitrile Nanofibrous Membranes

Ling Zhang [1], Junlu Sheng [1, 2,*], Yongbo Yao [1], Zhiyong Yan [1], Yunyun Zhai [3], Zhongfeng Tang [4] and Haidong Li [1]

[1] College of Materials and Textile Engineering, Nanotechnology Research Institute, Jiaxing University, Jiaxing 314001, China
[2] Key Laboratory of Yarn Materials Forming and Composite Processing Technology of Zhejiang Province, Jiaxing University, Jiaxing 314001, China
[3] Jiaxing Key Laboratory of Molecular Recognition and Sensing, College of Biological, Chemical Sciences and Engineering, Jiaxing University, Jiaxing 314001, China
[4] Shanghai Institute of Applied Physics, Chinese Academy of Sciences, Shanghai 201800, China;
* Correspondence: shengjunlu@126.com

Table S1. Atomic ratios of carbon, nitrogen, oxygen and silicon on the surface of PAN@AMP-1 nanofibrous membranes before breathability tests, PAN@AMP-1 nanofibrous membranes after breathability tests using pressures of 100 Pa, and PAN@AMP-1 nanofibrous membranes after breathability tests using pressures of 200 Pa. Data are calculated from XPS.

**Samples**	**Atomic percent (%)**			
	**C**	**O**	**N**	**Si**
PAN@AMP-1 nanofibrous membranes before breathability tests	52.38	23.48	2.16	21.98
PAN@AMP-1 nanofibrous membranes after breathability tests using pressures of 100 Pa	51.91	23.77	2.31	22.02
PAN@AMP-1 nanofibrous membranes after breathability tests using pressures of 200 Pa	52.02	23.61	2.41	21.95

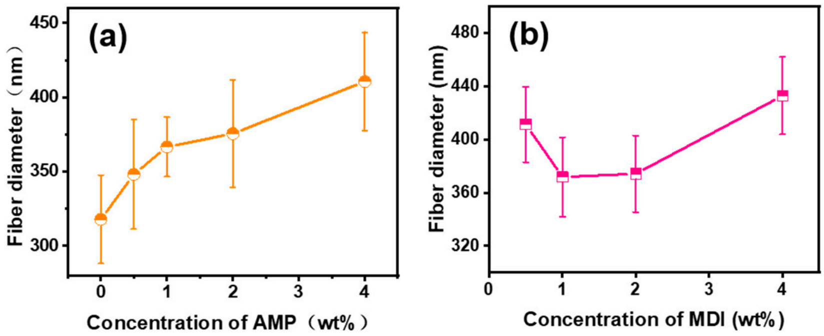

Figure S1. (a) Fiber diameters of PAN@AMP nanofibrous membranes modified with different concentrations of AMP. (b) Fiber diameters of PAN@AMP/MDI nanofibrous membranes modified with different concentrations of MDI.

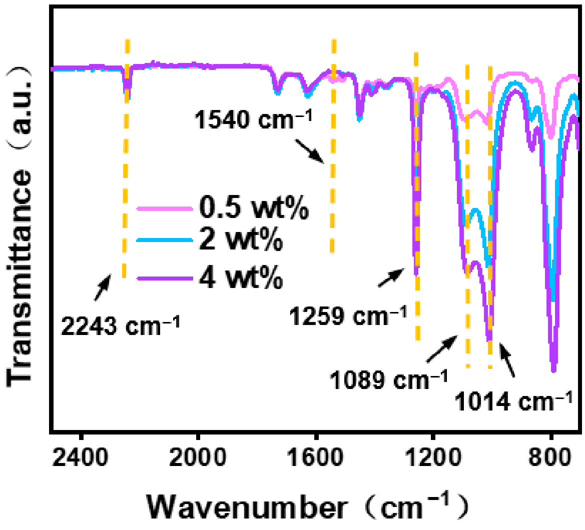

Figure S2. FT-IR spectra of hydrophobically modified PAN@AMP nanofibrous membranes with different concentrations of AMP.

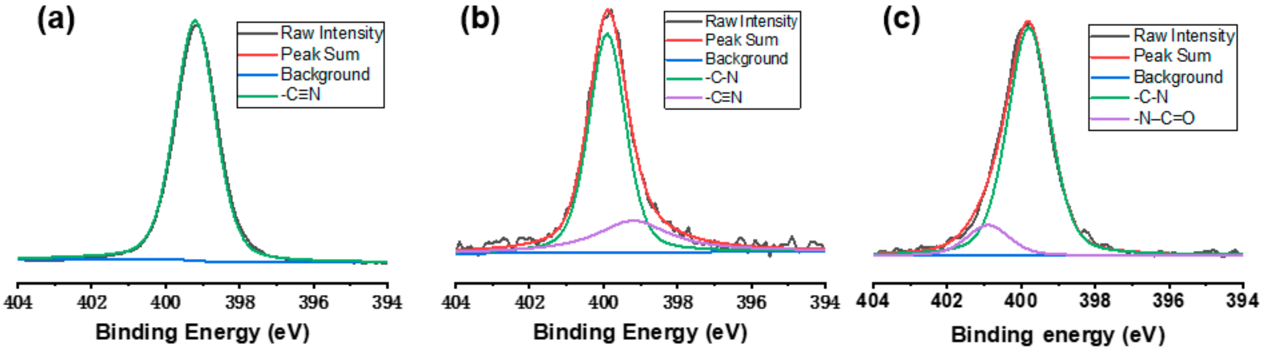

Figure S3. High-resolution XPS N1s spectra of (a) PAN original membranes, (b) PAN@AMP-1 nanofibrous membranes, and (c) PAN@AMP-1/MDI-2 nanofibrous membranes.
